# Returning aging cells to productivity

**DOI:** 10.7554/eLife.107777

**Published:** 2025-07-08

**Authors:** Rachana S Vaidya, Simon Y Tang

**Affiliations:** 1 Department of Orthopedic Surgery, Washington University in St. Louis St. Louis United States; 2 Departments of Orthopedic Surgery, Biomedical Engineering, and Mechanical Engineering and Materials Science, Washington University in St. Louis St. Louis United States

**Keywords:** senescence, platelet-derived growth factors (PDGFs), inflammation, intervertebral disc, Human

## Abstract

Platelet-derived growth factors can restore the proliferative potential of senescent cells taken from the degenerated intervertebral discs of aged humans.

**Related research article** Zhang C, Diaz-Hernandez ME, Fukunaga T, Sreekala S, Yoon ST, Haglund L, Drissi H. 2025. Therapeutic effects of PDGF-AB/BB against cellular senescence in human intervertebral disc. *eLife* **13**:RP103073. doi: 10.7554/eLife.103073.

Most people eventually suffer from low back pain. While many factors contribute to this pain, one of the most common culprits is the gradual breakdown of structures in the back called intervertebral discs (Diwan and Melrose, 2023). These discs have a unique jelly donut structure with a soft center, known as the nucleus pulposus, surrounded by tough outer rings of annulus fibrosus. Together, these structures absorb shocks and support movement (Swahn et al., 2024).

As we grow older, intervertebral discs lose water and structural integrity. Moreover, in addition to this mechanical decline, more and more cells enter a dysfunctional state called cellular senescence. Senescent cells are metabolically active, but the cycle cell has stopped, so they are unable to divide: instead, they persist in tissue and release a range of damaging molecules in a process known as the senescence-associated secretory phenotype (SASP).

Cellular senescence serves as a double-edged sword: it can protect the body in acute scenarios by amplifying inflammatory signals, but it can also drive degeneration when the accumulation of senescent cells reaches chronic levels. Senescence is caused by stressors like DNA damage, injury or oxidative stress, and it initially functions as a defense mechanism (Patil et al., 2018; Liu et al., 2020; Paramos-de-Carvalho et al., 2021). However, persistent senescent cells, particularly in aging or chronic disease contexts, become detrimental to our health by secreting inflammatory factors (such as cytokines, chemokines, and various tissue-degrading enzymes) that degrade tissues and perpetuate pathologies like osteoarthritis and disc degeneration (Jeon et al., 2018; Silwal et al., 2023).

Senescent cells can be targeted with drugs called senolytics that selectively eliminate them. In a small human trial, a short course of two senolytics (dasatinib and quercetin) reduced senescent cells in individuals with diabetic kidney disease (Hickson et al., 2019). In aging mice, the same drugs delayed disc degeneration and preserved tissue structure (Novais et al., 2021). However, senolytics have limitations in that their effects depend on timing, and they could have off-target effects on nearby healthy cells. This has spurred interest in drugs called senomorphics that reduce senescence without eliminating the senescent cells themselves, potentially offering a more targeted therapeutic approach (Wang et al., 2017; Le Pelletier et al., 2021; Zhang et al., 2023).

Now, in eLife, Hicham Drissi (Emory University and the Atlanta VA Medical Center) and colleagues – including Changli Zhang as first author – report that platelet-derived growth factors (PDGFs) can suppress senescent features in human intervertebral disc cells (Zhang et al., 2025). PDGFs are best known for their role in wound healing and tissue regeneration, and are abundant in platelet-rich plasma, a treatment that is already used in some clinics for back pain (Fiani et al., 2021).

The team – which also includes researchers from the Emory Orthopaedics & Spine Center and McGill University – isolated nucleus pulposus cells and annulus fibrosus cells from aged, degenerated human discs, and treated them with the AB and BB isoforms of PDGF. Using RNA sequencing, Zhang et al. found that the treatment suppressed genes involved in oxidative stress, mitochondrial dysfunction, and inflammation – which are all hallmarks of senescence. Meanwhile, genes involved in DNA repair and restarting the cell cycle were upregulated, indicating a shift toward a more proliferative, less inflammatory state (Figure 1).

**Figure 1.**
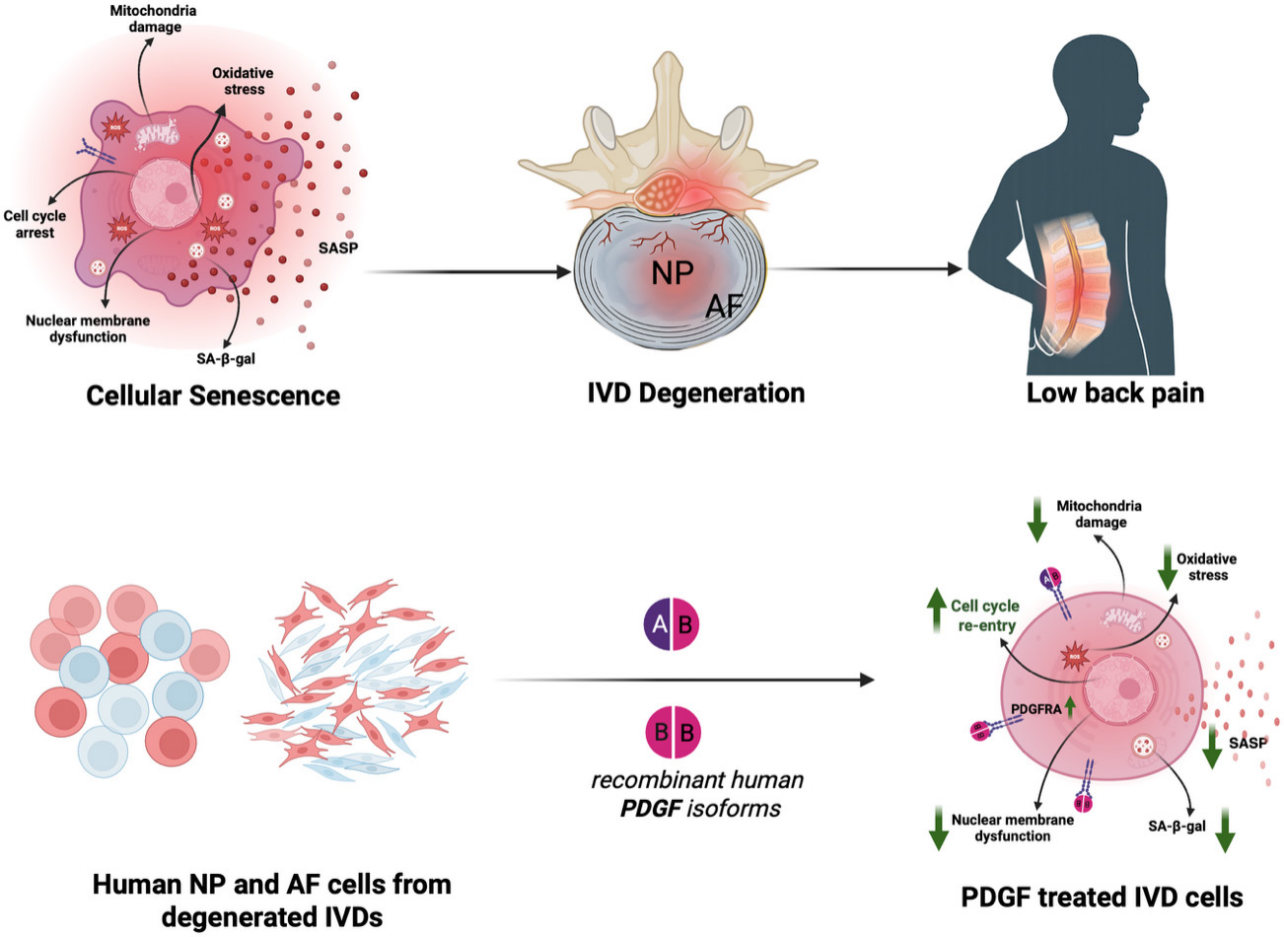
Using platelet-derived growth factors (PDGFs) to target cellular senescence and low back pain. (Top): Cellular senescence in the cells of the nucleus pulposus (NP) and the annulus fibrosus (AF) contributes to intervertebral disc (IVD) degeneration and low back pain. Senescent IVD cells (left) display mitochondrial damage, oxidative stress, a senescence-associated secretory phenotype (SASP), SA-β-gal activity, nuclear membrane dysfunction, and cell cycle arrest. These changes promote disc matrix breakdown and pain. (Bottom): Zhang et al. isolated NP cells (circles) and AF cells (elongated shapes) from degenerated human IVDs and treated them with isoforms of recombinant human PDGF (PDGF-AB and PDGF-BB). Treatment with PDGF reduced various markers of senescence (downward green arrows), while increasing production of PDGF receptor alpha (PDGFRA) and restarting the cell cycle. Healthy cells are shown in blue; senescent cells are shown in red.

Created with BioRender.

To validate these results, Zhang et al. created a model of stress-induced premature senescence by exposing healthy disc cells to high doses of X-rays. Subsequent treatment with the PDGF isoforms, particularly PDGF-BB, reduced classical markers of senescence. This treatment also increased the proportion of cells in the DNA synthesis phase, suggesting that PDGF helped to rescue these cells from permanent cell-cycle arrest.

Interestingly, the effects of PDGF treatment varied between the two compartments of the intervertebral disc. In nucleus pulposus cells, treatment increased levels of a receptor called PDGF receptor alpha and activated Wnt signaling, which helps preserve cell identity under stress. In annulus fibrosus cells, PDGF-BB downregulated genes involved in nerve growth, blood vessel formation, and mechanical strain, all processes that are linked to the pain associated with disc degeneration. While PDGF-BB has previously been shown to reduce cell death in intervertebral discs (Presciutti et al., 2014) and delay degeneration in rabbit model (Paglia et al., 2016), this is the first study to reveal its anti-senescent effects in human disc cells.

The effects of PDGF vary by its target cell type. Zhang et al. found that in aged or stressed disc cells, PDGF appears to restore cellular function by dampening pro-senescent pathways and promoting proliferation and repair, giving the cells in intervertebral discs a second chance. However, in mesenchymal cells, it transiently activates SASP-like genes without inducing senescence, likely as part of a normal repair response (Grigorieva et al., 2021). This highlights the need to understand cell-type-specific effects when designing senescence-targeted therapies.

These insights offer clues to how platelet-rich plasma could work in back pain therapy. In addition to easing pain, it might also help modulate senescent cells and restore disc health. Still, questions remain. Can PDGF reverse all forms of senescence, including those from telomere shortening? Can it be delivered effectively into the tissue of intervertebral discs? And how might it affect nearby cells, like immune cells or nerve endings? Answering these questions will require careful consideration and more research.

The most important takeaway from the work of Zhang et al. is that senescence may not always be a one-way street. Even in old, degenerated discs, some cells may be coaxed back into action. This raises the exciting possibility that chronic back pain may not be inevitable. With the right molecular signals, even aging discs can be productive again.
